# Rumen fermentation, methane concentration, and blood metabolites of cattle receiving dietetical phytobiotic and cobalt (II) chloride

**DOI:** 10.14202/vetworld.2022.2551-2557

**Published:** 2022-11-11

**Authors:** Vitaliy Ryazanov, Galimzhan Duskaev, Elena Sheida, Baer Nurzhanov, Marina Kurilkina

**Affiliations:** Federal Research Centre of Biological Systems and Agrotechnologies, RAS, 460000, Orenburg, Russia

**Keywords:** *Artemisia absinthium* herbal, cobalt (II) chloride, digestibility, methane, nitrogen, volatile fatty acids

## Abstract

**Background and Aim:**

Ensuring the genetic potential of ruminants through nutrition studies using medicinal plants and trace element metals is an urgent task. This study aimed to study the effect of *Artemisia absinthium* L. (Asteraceae) herb plant separately and in combination with cobalt (II) chloride (CoCl_2_) chelate compounds on the course of metabolic processes in the rumen, methane concentration, and biochemical blood parameters in bulls.

**Materials and Methods:**

Control group (BD: Basal diet); experimental Group I – BD + *A. absinthium* herb at a dose of 2.0 g/kg dry matter (DM), experimental Group II – BD + *A. absinthium* herb at a dose of 2.0 g/kg DM + CoCl_2_ (1.5 mg/kg), and experimental Group III – BD + CoCl_2_ were set (1.5 mg/kg). The study was conducted on 16 beef bulls (Kazakh white-headed breed) aged 13–14 months, with an average live weight of 330–335 kg. Enzymatic processes in the rumen were studied, including the level of volatile fatty acids (using the gas chromatography method), nitrogen and its fractions (using the Kjeldahl method), methane concentration, and morphological and biochemical blood composition.

**Results:**

There was a decrease in the concentration of acetic acid in experimental group I (15.9%) (p < 0.05) and in the III group (60.3%) and propionic acid in all experimental groups by 5.6%–47.3% (p < 0.05). Feeding *A. absinthium* herb as part of the diet of experimental Group I contributed to a decrease in methane concentration by 17.8% (p = 0.05) and the lowest methane concentration was noted for experimental Group III. It was less than in control by 59.1% (p < 0.05). An increase in the concentration of glucose, total protein, and creatinine was found in the experimental groups (p < 0.05). The digestibility of organic matter (3.5%), crude fiber (3.6%), and hemicellulose (11.0%) increased with the feeding of *A. absinthium* herb.

**Conclusion:**

Thus, using biocomplexes based on *A. absinthium* herb and CoCl_2_ do not harm the rumen fermentation of cattle. Still, further microbiome studies are required to evaluate the effects of *A. absinthium* on cattle properly.

## Introduction

An increase in beef production is associated with increased loads on the digestive tract of the animal, which leads, among other things, to various diseases. In this regard, cattle raising is closely related to the study of enzymatic processes in the rumen [1]. The improvement in metabolic processes in rumen is possible using phytobiotics [2]. Their use can contribute to the inhibition of methane formation as one of the factors of energy loss by the body of the animal and can reduce the effect of greenhouse gases [3]. At the same time, phytobiotics are capable of stimulating the microorganisms of the rumen by improving their digestion and use of structural carbohydrates [4] with the formation of more carboxylic acids [5]. Thus, by increasing rumen activity in cattle with feed additives based on plants containing various biologically active substances, there is an improvement in feed digestibility [6].

In addition, the use of phytobiotics leads to the rejection of antibiotics and other chemicals in the treatment and prevention of microbial resistance [7]. There are also positive data on the use of phytochemicals combined with probiotics to modulate the animal digestive system [8, 9]. However, there is limited information on the use of biocomplexes based on phytobiotics and trace elements of metals [10, 11]. Trace elements affect the concentration of volatile fatty acids, the pH of the rumen, methane formation, and fermentation in the rumen of ruminants [12]. Adding cobalt to animal diets improves the digestibility of feed fiber in the rumen [13], increases the immunity of animals [14], and increases the concentration of B12 vitamin [15].

Thus, the issue of studying biocomplexes based on phytonutrients and trace element metals to regulate metabolism in the rumen of cattle remains open and requires further research. This study aimed to study the effect of *Artemisia absinthium L. (Asteraceae)* herb plant separately and in combination with cobalt (II) chloride (CoCl_2_) chelate compounds on the course of metabolic processes in the rumen, methane concentration, and biochemical blood parameters in bulls.

## Materials and Methods

### Ethical approval and informed consent

The ethical approval of this study was received from the ethics committee of the Federal Research Center of Biological Systems and Agrotechnologies of the Russian Academy of Sciences. Informed consent was obtained from all participants before the study.

### Study period and location

The study was conducted from August 2021 to May 2022, in the conditions of the physiological yard of the Department of Feeding Farm Animals and Feed Technology named after Professor S.G. Leushin of the Federal State Research University “Federal Scientific Center for Biological Systems and Agrotechnologies of the Russian Academy of Sciences”.

### *In vivo* method

The research was conducted under *in vivo* conditions (“Federal Research Center of Biological Systems and Agrotechnologies of the Russian Academy of Sciences”). Objects of research: 16 beef bulls (Kazakh white-headed breed) aged 13–14 months, with an average live weight of 330–335 kg. During the study, vegetative parts of *A. absinthium* herb plant collected in 2021 during the flowering period (July–August) were used. The vegetative parts of *A. absinthium* were dried in a dark room at room temperature (20°C). Cobalt (II) chloride (CoCl_2_) (manufacturer: LLC NPK “Ascont+,” Moscow region, Russia), in the form closest to the natural one associated with amino acids and peptides, is a cofactor in enzymes that play an essential role in the protective bodily function of animals, growth, and reproduction. The animals were fitted with rumen fistulas (ANKOM Technology, d = 80 mm), and every effort was made to reduce the harm.

The experiment was conducted in four repetitions using a 4 × 4 Latin square. The diet of all animals, especially those in the control group, consisted of legume hay 32.6%, mixed hay 47.4%, grain mixture 19.0%, and 1.0% mineral supplements (Basal diet (BD); Table-1) [16]. The animals had free access to water. Animals in experimental Group I were given *A. absinthium* herb at a dose of 2.0 g/kg dry matter (DM) in addition to BD. Animals in experimental Group II received *A. absinthium* herb at a dose of 2.0 g/kg DM and CoCl_2_ (1.5 mg/kg) in addition to BD. Animals in experimental Group III were given CoCl_2_ (1.5 mg/kg) in addition to BD. The preparatory period was 20 days, and the accounting period was 20 days. The consumed feed and excreted feces were considered during the experiment, and an average sample was taken. The feed and feces were subjected to chemical analysis (e.g., DM, crude protein, crude fat, crude fiber, crude ash, organic matter, calcium, phosphorus, nitrogen-free extractives, hemicellulose, neutral-detergent fiber, and acid-detergent fiber).

**Table-1:** The general composition of the diet for all groups of animals, % [16].

Component	Content
Mixed grass hay, %	47.4
Legume hay, %	32.6
Grain mixture, %	19.0
Mineral, %	1.0
Total	100
The diet contains (% DM)	
Dry matter	94.7
Crude protein	5.9
Crude fiber	36.75
Neutral detergent fiber	63.12
Acid detergent fiber	46.51
Hemicellulose	16.61
Crude fat	2.73
Organic matter	93.4
Calcium	0.51
Phosphorus	0.37
Crude ash	1.28
Nitrogen-free extractives	53.8

DM=Dry matter

The digestibility coefficients of the feed were determined by considering the digested part as the total amount of nutrients consumed with the feed, expressed as a percentage. At the end of each accounting period, animal blood was taken from the jugular vein into vacuum tubes with a coagulation activator and separation gel (Zhejiang Gongdong Medical Technology Co., Ltd., China) with a volume of 6 mL. The analysis was conducted on devices such as the CS-T240 automatic biochemical analyzer (Manufacturer: DIRUI Industrial Co. Ltd., China) and automatic veterinary hematology analyzer DF50 Vet (Dymind, China).

### Chemical analysis

At the end of each accounting period, the rumen content was selected to determine volatile fatty acids by gas chromatography (Chromatec-Crystal 5000, CJSC SKB “Chromatec,” Russia) on a chromatograph with a flame ionization detector and a capillary column. The parameters were set as follows: A programmable temperature increase of the column thermostat from 60°C to 260°C, the temperature of the injector 250°C, and the detector 250°C. The necessary gas velocities were selected. The analysis time was 40 min, and the sample input was 1 mm^3^. Solutions of acid mixtures with concentrations of 10, 25, and 50 mg/cm^3^ were used as samples for calibration. At least two chromatograms of each solution were recorded, starting with a lower concentration. The determination of nitrogen forms was conducted on Millab equipment (Millab, Italy) using the Kjeldahl method, consisting of three stages. The first stage involved mineralizing the sample with concentrated sulfuric acid, potassium sulfate, and copper sulfate. In this case, organic matter was oxidized in the presence of a catalyst to CO_2_ and H_2_O, and all nitrogen passed into ammonia and bound to sulfuric acid, forming ammonium sulfate. An alkali solution neutralized excess sulfuric acid to release ammonia. The second stage involved the distillation of ammonia into a receiving flask with a standard boric acid solution. The third stage was determining the mass fraction of nitrogen by titration. The methane concentration was calculated according to the formula proposed earlier [17].

### Statistical analysis

The data from this study were analyzed using Statistical Package for the Social Sciences version 21.0 (IBM, USA). The mean (M), standard deviations (± σ), and standard deviation errors (± SE) were calculated. A non-parametric analysis method was used to compare the variants (Student’s t-criterion). The differences were statistically significant at p < 0.05 and p < 0.01 [18].

## Results

The structure and nutritional composition of the diet for the control and experimental groups were the same throughout the study.

According to the experiment results, a decrease in the digestibility of DM in experimental Group I was found by 0.6% (p < 0.05) compared with the control. The hemicellulose digestibility coefficient in experimental Group I was higher by 11.0% relative to the control group and by 10.2%–12.1% (p < 0.05) in experimental Groups II and III, respectively. The digestibility of neutral detergent fiber in the first experimental group decreased by 0.4% relative to the control group (p < 0.05), and acid detergent fiber decreased by 0.7% (p < 0.05). The digestibility coefficients of the nitrogen-free extractives were higher in all experimental groups relative to the control (1.7%–5.8% [p < 0.05]), as shown in Table-2. The digestibility of organic matter was higher by 2.1% and 6.1% in experimental Groups II and III (p < 0.05) than in the control group. The digestibility of DM in experimental Group III increased by 1.5% (p < 0.05) and crude fiber increased by 2.0% (p < 0.05) relative to the control. The digestibility coefficients of neutral detergent fiber and acid detergent fiber in experimental Group III increased by 1.3%–2.9% (p < 0.05) and hemicellulose by 0.8%–2.1% (p < 0.05) (Table-2).

**Table-2:** Coefficients of digestibility of nutrients among the groups %.

Indicator	Groups			

	Control	Experimental I	Experimental II	Experimental III
Dry matter	69.0 ± 0.57	68.4 ± 0.62^a^	67.5 ± 0.65	70.1 ± 0.84^ac^
Organic matter	68.6 ± 0.83	71.2 ± 0.73^a^	70.1 ± 0.67^a^	73.1 ± 0.81^ab^
Crude protein	75.3 ± 0.69	64.7 ± 0.67	67.2 ± 0.59^b^	67.2 ± 0.63
Crude fat	65.2 ± 1.03	65.0 ± 0.92	51.6 ± 1.1	60.6 ± 0.78
Crude fiber	67.2 ± 0.75	69.8 ± 0.82^a^	66.6 ± 0.88	68.6 ± 0.89^ac^
Neutral detergent fiber	74.5 ± 0.82	74.2 ± 0.75	73.4 ± 0.74	75.6 ± 0.78^abc^
Acid detergent fiber	73.0 ± 0.67	72.5 ± 0.71^a^	71.8 ± 0.69	74.0 ± 0.67^abc^
Hemicellulose	79.0 ± 0.88	88.8 ± 0.81^a^	78.0 ± 0.78	79.7 ± 0.84^ac^
Nitrogen-free extractives	70.2 ± 0.71	71.4 ± 0.75^a^	71.5 ± 0.80^ab^	74.6 ± 0.79^abc^

a p<0.05 when compared with the control;

b p<0.05 when compared with Group I,

^c^p<0.05 when compared with Group II; Experimental I=*Artemisia absinthium* herbal; Experimental II=*Artemisia absinthium* herbal+CoCl_2_; Experimental III=CoCl_2_

There was a decrease in the concentration of acetic acid in experimental Group I by 15.9% (p < 0.05) and in Group III by 60.3% (p < 0.05) relative to the control, against the background of its increase in experimental Group II, which increased by 9.0% (p < 0.05). The concentration of propionic acid decreased in all experimental groups. The concentration decreased by 5.6%–47.3% (p < 0.05) (Table-3).

**Table-3:** Concentration of volatile fatty acids in the rumen fluid *in vivo* when using various combinations of metal trace element phytobiotic, mol/L.

Group	Volatile fatty acids				

	Acetic	Propionic	Butanoic	Valerian	Caproic
Control	0.864 ± 0.004	0.550 ± 0.002	0.553 ± 0.007	0.177 ± 0.003	0.091 ± 0.012
I experienced	0.726 ± 0.006*	0.468 ± 0.004*	0.448 ± 0.006*	0.050 ± 0.013	0.036 ± 0.014*
II experienced	0.950 ± 0.003*	0.519 ± 0.001**	0.532 ± 0.007	0.097 ± 0.008	0.062 ± 0.013*
III experienced	0.343 ± 0.007*	0.290 ± 0.008*	0.282 ± 0.011	0.026 ± 0.013	0.016 ± 0.007

* p<0.05;

* p<0.01; Experimental I=*Artemisia absinthium* herbal; Experimental II=*Artemisia absinthium* herbal+CoCl_2_; Experimental III=CoCl_2_

There was an increase in total nitrogen in experimental Group III by 19.2% (p < 0.05), and the protein form of nitrogen increased by 31.0% (p < 0.05) in comparison with the control. Non-protein nitrogen fractions in experimental Group II were more significant than the other groups by 25.9%–49.2% (p < 0.05). A decrease in the ammonia form of nitrogen concentration by 25.0% (p < 0.05) was noted in experimental Group II compared to the control, as shown in Table-4.

**Table-4:** The content of nitrogenous compounds in the rumen fluid *in vivo*, mg/%.

Group	Forms of nitrogen				

	Total	Non-protein	Ammonia urea	Urea	Protein
Control	100.1 ± 1.7	28.0 ± 1.1	1.4 ± 0.9	2.63 ± 1.5	72.1 ± 2.0
I experienced	71.4 ± 1.9	28.0 ± 1.4	1.4 ± 0.2	1.88 ± 1.7*	43.4 ± 1.8*
II experienced	94.5 ± 0.8**	37.8 ± 2.1*	1.05 ± 0.1*	2.63 ± 1.4	56.7 ± 1.3*
III experienced	123.9 ± 1.4*	19.2 ± 1.6*	1.75 ± 0.3*	1.5 ± 0.6*	104.6 ± 1.5*

* p<0.05;

** p<0.01; Experimental I=*Artemisia absinthium* herbal; Experimental II=*Artemisia absinthium* herbal+CoCl_2_; Experimental III=CoCl_2_

Feeding *A. absinthium* herb as part of the diet of the experimental Group I contributed to a decrease in methane concentration by 17.8% (p < 0.05). The combination of *A. absinthium* herb and chelated cobalt chloride compounds in Group II revealed an increase in concentration by 7.7% (p < 0.05) compared with the control. The lowest concentration was recorded in experimental Group III, which was lower than that in the control group by 59.1% (p < 0.05), as shown in Table-5.

**Table-5:** Production of methane (CH_4_) in the rumen fluid *in vivo*, mol/L.

Indicator	Group			

	Control	Experimental I	Experimental II	Experimental III
CH_4_ (mol/L)	0.4588 ± 0.039	0.3772 ± 0.028*	0.4976 ± 0.017*	0.1874 ± 0.021*
CO_2_ e/g	185.0 ± 1.2	151.25 ± 2.3*	197.5 ± 1.8	75.0 ± 1.6*

* p<0.05; СО_2_ е/g – carbon dioxide equivalent, CO_2_e; Experimental I=*Artemisia absinthium* herbal; Experimental II=*Artemisia absinthium* herbal+CoCl_2_; Experimental III=CoCl_2_

Concerning the blood analysis of experimental animals, it was found that the increase in glucose concentration in all experimental groups ranged by 4.0%–19.1% (p < 0.05) compared with the control. An increase in the total protein content in the blood was also noted for all experimental groups (p < 0.05), as shown in Table-6.

**Table-6:** Biochemical parameters of cattle blood.

Indicator	Group			

	Control	Experimental I	Experimental II	Experimental III
Glucose, mmol/L	3.81 ± 0.03	3.97 ± 0.05*	4.0 ± 0.04*	4.71 ± 0.09*
Total protein, g/L	67.65 ± 0.08	111.3 ± 0.17*	115.0 ± 0.12*	96.07 ± 0.11*
Albumin, g/L	35.0 ± 0.06	36.0 ± 0.04*	37.0 ± 0.05*	34.0 ± 0.08
ALT, Ed/L	36.1 ± 0.01	30.2 ± 0.06*	36.9 ± 0.07*	45.0 ± 0.05*
AST, Ed/L	72.7 ± 0.07	70.2 ± 0.06	81.5 ± 0.08*	99.3 ± 0.11*
Total bilirubin, mmol/L	2.79 ± 0.05	2.58 ± 0.11	0.47 ± 0.07	2.15 ± 0.07
Straight bilirubin, mmol/L	3.12 ± 0.04	1.68 ± 0.09	1.14 ± 0.09	1.5 ± 0.06
Cholesterol, mmol/L	2.03 ± 0.03	2.39 ± 0.04*	2.36 ± 0.06*	2.94 ± 0.08*
Triglycerides, mmol/L	0.1 ± 0.04	0.17 ± 0.03	0.06 ± 0.002	0.29 ± 0.1
Urea, mmol/L	2.0 ± 0.06	2.5 ± 0.05*	1.8 ± 0.03*	1.3 ± 0.04*
Creatinine, mmol/L	109.7 ± 0.18	249.4 ± 0.27*	114.2 ± 0.15*	147.4 ± 0.31*
Uric acid, mmol/L	2.9 ± 0.05	2.4 ± 0.06*	1.0 ± 0.02	3.43 ± 0.08*
Iron, mmol/L	20.8 ± 0.04	19.4 ± 0.05	24.2 ± 0.07	32.8 ± 0.09
Magnesium, mmol/L	0.87 ± 0.03	0.78 ± 0.02	0.74 ± 0.04	1.15 ± 0.05
Calcium, mmol/L	2.25 ± 0.02	2.32 ± 0.03	2.54 ± 0.04	3.2 ± 0.07
Phosphorus, mmol/L	4.19 ± 0.04	0.57 ± 0.06	0.56 ± 0.05	1.36 ± 0.12

* p<0.05; ALT=Alanine aminotransferase, AST=Aspartate aminotransferase, Experimental I=*Artemisia absinthium* herbal; Experimental II=*Artemisia absinthium* herbal+CoCl_2_; Experimental III=CoCl_2_

The combined and separate use of *A. absinthium* herb and chelated compounds of cobalt chloride in experimental Groups I and II resulted in an increase in the water-soluble albumin protein by 2.7% (p < 0.05) and 5.4% (p < 0.05) in comparison with the control. The level of aspartate aminotransferases in the blood was increased in experimental Group II by 10.7% (p < 0.05) and in experimental Group III by 26.7% (p < 0.05) compared with the control.

The level of alanine aminotransferase increased most significantly in experimental Group III. In the experimental groups, blood cholesterol increased by 15.0%–30.9% (p < 0.05). The concentration of breakdown products of proteins synthesized by the liver and urea decreased in experimental Groups II and III by 10.0% and 35.0%, respectively, compared with the control group. The creatinine level, which characterizes the final stage of protein breakdown in muscle tissue and liver, increased in all experimental groups. Hence, the difference with the control group was 3.9%–56.0% (p < 0.05), as shown in Table-6.

## Discussion

The stimulation of enzymatic processes in the rumen due to the rich content of biologically active substances saponins, tannins, essential oils, and flavonoids was promoted using phytomaterials [7, 19], which have antimicrobial and anti-inflammatory properties. Phytomaterials increase the appetite and contractile motility of the scar walls, improve the process of protein formation, reduce methane emission [20], and restore rumen pH without reducing the digestibility of nutrients in the diet.

We also observed an increase in the digestibility of structural carbohydrates in terms of hemicellulose in experimental Group I using *A. absinthium* herb by an average of 11.0% compared to the control and experimental Groups II and III. At the same time, a slight deviation in the digestion of neutral detergent and acid detergent fibers was noted in experimental Groups I and II. Some *Artemisia* species are known to be able to improve the enzymatic characteristics of the rumen by increasing the digestibility of DM and neutral detergent fiber. Perhaps, this effect is characterized by the selective composition of essential oils that are part of plants [6, 21].

In contrast, the presence of cobalt-chelated compounds increased the digestibility of neutral detergent and acid detergent fiber, also related to difficult-to-hydrolyze natural polymers [22]. A decrease in methane formation was also observed, which is consistent with our results. The smallest amount of methane was detected in experimental Group I treated only with *A. absinthium* herb, and in Group III, of animals treated with cobalt. In a previous study [23], the therapeutic properties of cobalt salts were reported, indicating the activity of cobalt in the digestive tract and showing that the formation of Vitamin B12 is not possible without the participation of cobalt. In turn, cobalt is necessary for metabolic reactions involved in the metabolism of adenosylcobalamin and methylcobalamin, which are coenzymes of methylmalonyl coenzyme A mutases, and methionine synthetases are also crucial in obtaining energy through metabolism in the rumen [15]. A positive relationship was observed between the formation of Vitamin B12 and the concentration of neutral detergent fibers and acid detergent fibers in the diet observed [24, 25]. In our experience, complex polymer compounds dominated the diet significantly. As reported by Brewer *et al*. [26], various cobalt compounds are metabolized differently by the body.

It is known that the use of phytomaterials increased the total amount of volatile fatty acids [27]. In our experiment, the high values of volatile fatty acids were noted when using *A. absinthium* herb in combination with cobalt chelate compounds in experimental Group II. The activity of microorganisms capable of transforming the urea form of nitrogen to ammonia was indicated by the presence of microbial proteins [28]. In our work, adding *A. absinthium* herb in the experimental Group I reduced the urea form of nitrogen concentration. At the same time, introducing only cobalt in the experimental group III contributed to a decrease in the urea form of nitrogen and an increase in ammonia, with a simultaneous increase in protein fractions.

Introducing phytonutrients and trace elements of metals into feed affect the formation of blood metabolites. Blood metabolites characterizing the energy balance of the body of the animal include glucose, fructosamine, insulin, non-esterified fatty acids, β-hydroxybutyrate, cholesterol, total protein, and albumin [29]. In our study, the glucose concentration increased in the experimental groups. This may be explained by the antidiabetic mechanism of the action of substances that are part of *Artemisiae* species [30], such as *Artemisia dracunculus* L. extract, which improves glucose homeostasis [31].

High concentrations of energy in the body of an animal are associated with a high concentration of glucose in the blood [32]. The total index of the protein fractions was higher than that of the control in all experimental groups. Albumin concentrations were higher in experimental Groups I and II, which received *A. absinthium* herb separately and combined with cobalt chelate compounds. This is probably due to the ability of albumins to bind actively to biologically active substances that are part of *Artemisiae* [33]. In experimental Group III, which received only the cobalt compound, there was a decrease in albumin, which was also found in the previous study [34].

The use of new feed additives in the nutrition of polygastric animals require much attention to abandon antibiotics in animal husbandry while stimulating the digestive system. The use of phytobiotics separately or combined with trace elements of metals in so-called biocomplexes is of broad scientific interest.

## Conclusion

Thus, using biocomplexes based on A. absinthium herb (2/0 g/kg DM) and CoCl_2_ (1.5 mg/kg) does not affect fermentation in the rumen of cattle. Still, further microbiome studies are required to evaluate the effects of *A. absinthium* on cattle.

## Authors’ Contributions

GD: Conception and design of the study. VR: Coordinated the analysis. ES and MK: The conclusive and final remarks. GD, VR, and BN: Drafted and revised the manuscript. All authors have read and approved the final manuscript.
